# Information Entropy Algorithms for Image, Video, and Signal Processing

**DOI:** 10.3390/e23080926

**Published:** 2021-07-21

**Authors:** Gwanggil Jeon

**Affiliations:** Department of Embedded Systems Engineering, College of Information Technology, Incheon National University, 119 Academy-ro, Yeonsu-gu, Incheon 22012, Korea; gjeon@inu.ac.kr

Information entropy is a basic concept in information theory associated with any random variable. Information entropy can be interpreted as the average level of information, surprise, and uncertainty inherent in a variable’s possible outcomes. The concept of information entropy was introduced by Claude Shannon in his 1948 paper, “A Mathematical Theory of Communication”. Over the last few years, entropy has become an adequate trade-off measure in image, video, and signal processing. In particular, entropy measures have been used in the image, video, and signal processing area to cover the topics of Chroma subsampling, coding tree unit, color space, compression artifact, image resolution, and macroblock pixel. In addition, entropy measures have also been used in video processing to cover the topics of bit rate, display resolution, frame rate, interlaced video, and video quality. As the daily produced data are increasing rapidly, more effective applications for image, video, and signal processing are required.

In light of these and many other challenges, a special issue of “Information Entropy Algorithms for Image, Video, and Signal Processing” has been dedicated to address the current status, challenges, and future research priorities for the entropy of signal processing.

Starting from the above considerations, this special issue aims to investigate the impact of the adoption of advanced and innovative information entropy-based algorithms in image, video, signal processing applications, including the ones that take advantage of recent big data, compression, multichannel, sensor, and prediction techniques. This edition of the special issue is focused primarily on signal processing for image and video applications with special emphasis on stream processing and imaging platforms. This issue is intended to provide a highly recognized international forum to present recent advances in *Entropy*. We welcomed both theoretical contributions as well as papers describing interesting applications. Papers were invited for this special issue considering aspects of this problem, including:

- Multichannel imaging;
- Sensor size, channel number, dynamic range;
- Modeling of signal processing;
- Compression approach;
- Entropy-based video coding;
- Prediction and redundancy for video coding;
- Noise removal approach;
- GPU-based methods for signal processing;
- Signal quality assessment.

After review, a total of nine papers have been accepted for publication in this issue.

At present, many Deep Neural Network (DNN) methods have been widely used for hyperspectral image classification. Promising classification results have been obtained by utilizing such models. However, due to the complexity and depth of the model, increasing the number of model parameters may lead to an overfitting of the model, especially when training data are insufficient. As the performance of the model mainly depends on sufficient data and a large network with reasonably optimized hyperparameters, using DNNs for classification requires better hardware conditions and sufficient training time. The contribution by Xu et al. [1], “A Hyperspectral Image Classification Approach Based on Feature Fusion and Multi-Layered Gradient Boosting Decision Trees” proposes a feature fusion and multi-layered gradient boosting decision tree model (FF-DT) for hyperspectral image classification. First, the authors fuse extended morphology profiles (EMPs), linear multi-scale spatial characteristics, and nonlinear multi-scale spatial characteristics as final features to extract both special and spectral features. Furthermore, a multi-layered gradient boosting decision tree model is constructed for classification. The authors conduct experiments based on three datasets, which in this paper are referred to as the Pavia University, Indiana Pines, and Salinas datasets. It is shown that the proposed FF-DT achieves better performance in classification accuracy, training conditions, and time consumption than other current classical hyperspectral image classification methods.

Chaos-based encryption has been shown to play an increasingly important and dominant role in modern multimedia cryptography compared with traditional algorithms. The contribution by Yasser et al. [2], “A Chaotic-Based Encryption/Decryption Framework for Secure Multimedia Communications” proposes novel chaotic-based multimedia encryption schemes utilizing 2D alteration models for high secure data transmission. A novel perturbation-based data encryption for both confusion and diffusion rounds is proposed. Their chaotification structure is hybrid, in which multiple maps are combined for media encryption. Blended chaotic maps are used to generate the control parameters for the permutation (shuffling) and diffusion (substitution) structures. The proposed schemes not only maintain great encryption quality reproduced by chaotic maps, but also possess other advantages, including key sensitivity and low residual clarity. Extensive security and differential analyses documented that the proposed schemes are efficient for secure multimedia transmission; as well, the encrypted media possess resistance to attacks. Additionally, statistical evaluations using well-known metrics for specific media types show that proposed encryption schemes can acquire low residual intelligibility with excessive nice recovered statistics. Finally, the advantages of the proposed schemes have been highlighted by comparing them against different state-of-the-art algorithms from literature. The comparative performance results documented showed that their schemes are extra efficacious when compared to their data-specific counterpart methods.

Branch length similarity (BLS) entropy is defined in a network consisting of a single node and branches. In the contribution by Lee and Park [3], “Novel Features for Binary Time Series Based on Branch Length Similarity Entropy,” authors mapped the binary time-series signal to the circumference of the time circle so that the BLS entropy can be calculated for the binary time-series. The authors obtained the BLS entropy values for “1” signals on the time circle. The set of values are the BLS entropy profile. They selected the local maximum (minimum) point, slope, and inflection point of the entropy profile as the characteristic features of the binary time-series and investigated and explored their significance. The local maximum (minimum) point indicates the time at which the rate of change in the signal density becomes zero. The slope and inflection points correspond to the degree of change in the signal density and the time at which the signal density changes occur, respectively. Moreover, the authors show that the characteristic features can be widely used in binary time-series analysis by characterizing the movement trajectory of Caenorhabditis elegans. They also mention the problems that need to be explored mathematically in relation to the features and propose candidates for additional features based on the BLS entropy profile.

The question of beauty has inspired philosophers and scientists for centuries. Today, the study of aesthetics is an active research topic in fields as diverse as computer science, neuroscience, and psychology. Measuring the aesthetic appeal of images is beneficial for many applications. In the contribution by Khalili et al. [4], “An Information Theory Approach to Aesthetic Assessment of Visual Patterns,” authors study the aesthetic assessment of simple visual patterns. The proposed approach suggests that aesthetically appealing patterns are more likely to deliver a higher amount of information over multiple levels in comparison with less aesthetically appealing patterns when the same amount of energy is used. The proposed approach is evaluated using two datasets; the results show that the proposed approach is more accurate in classifying aesthetically appealing patterns compared to some related approaches that use different complexity measures.

The world has faced a coronavirus outbreak, which, in addition to lung complications, has caused other serious problems, including cardiovascular issues. There is still no explanation for the mechanisms of coronavirus that trigger dysfunction of the cardiac autonomic nervous system (ANS). In the contribution by Bajić et al. [5], “Entropy Analysis of COVID-19 Cardiovascular Signals,” authors believe that the complex mechanisms that change the status of ANS could only be solved by advanced multidimensional analysis of many variables, obtained both from the original cardiovascular signals and from laboratory analysis and detailed patient history. The aim of this paper is to analyze different measures of entropy as potential dimensions of the multidimensional space of cardiovascular data. The measures were applied to heart rate and systolic blood pressure signals collected from 116 patients with COVID-19 and 77 healthy controls. Methods that indicate a statistically significant difference between patients with different levels of infection and healthy controls will be used for further multivariate research. As a result, it was shown that a statistically significant difference between healthy controls and patients with COVID-19 was shown by sample entropy applied to integrated transformed probability signals, common symbolic dynamics entropy, and copula parameters. Statistical significance between seriously and mildly affected patients with COVID-19 can only be achieved by cross-entropies of heart rate signals and systolic pressure. This result contributes to the hypothesis that the severity of COVID-19 disease is associated with ANS disorder and encourages further research.

Uncertainty is at the heart of decision-making processes in most real-world applications. Uncertainty can be broadly categorized into two types: aleatory and epistemic. Aleatory uncertainty describes the variability in the physical system where sensors provide information (hard) of a probabilistic type. Epistemic uncertainty appears when the information is incomplete or vague, such as judgments or human expert appreciations in linguistic form. Linguistic information (soft) typically introduces a possibilistic type of uncertainty. The contribution by Solaiman et al. [6], “A New Hybrid Possibilistic-Probabilistic Decision-Making Scheme for Classification” is concerned with the problem of classification where the available information, concerning the observed features, may be of a probabilistic nature for some features, and of a possibilistic nature for some others. In this configuration, most encountered studies transform one of the two information types into the other form, and then apply either classical Bayesian-based or possibilistic-based decision-making criteria. In this paper, a new hybrid decision-making scheme is proposed for classification when hard and soft information sources are present. A new Possibilistic Maximum Likelihood (PML) criterion is introduced to improve classification rates compared to a classical approach using only information from hard sources. The proposed PML allows one to jointly exploit both probabilistic and possibilistic sources within the same probabilistic decision-making framework, without imposing the conversion of the possibilistic sources into probabilistic ones, and vice versa.

The probability density function (PDF) valid for the Gaussian case is often applied for describing the convolutional noise pdf in the blind adaptive deconvolution problem, although it is known that it can be applied only at the latter stages of the deconvolution process, where the convolutional noise pdf tends to be approximately Gaussian. Recently, the deconvolutional noise pdf was approximated with the Edgeworth Expansion and with the Maximum Entropy density function for the 16 Quadrature Amplitude Modulation (QAM) input but no equalization performance improvement was seen for the hard channel case with the equalization algorithm based on the Maximum Entropy density function approach for the convolutional noise pdf compared with the original Maximum Entropy algorithm, while for the Edgeworth Expansion approximation technique, additional predefined parameters were needed in the algorithm. In the contribution by Shlisel and Pinchas [7], “Improved Approach for the Maximum Entropy Deconvolution Problem,” the Generalized Gaussian density (GGD) function and the Edgeworth Expansion are applied for approximating the convolutional noise pdf for the 16 QAM input case, with no need for additional predefined parameters in the obtained equalization method. Simulation results indicate that improved equalization performance is obtained from the convergence time point of view of approximately 15,000 symbols for the hard channel case with their new proposed equalization method based on the new model for the convolutional noise pdf compared to the original Maximum Entropy algorithm. By convergence time, the authors mean the number of symbols required to reach a residual inter-symbol-interference (ISI), for which reliable decisions can be made on the equalized output sequence.

The Gerchberg–Saxton (G-S) algorithm is a phase retrieval algorithm that is widely used in beam shaping and optical information processing. However, the G-S algorithm has difficulty obtaining the exact solution after iterating, and an approximate solution is often obtained. In the contribution by Zhao et al. [8], “Modified Gerchberg–Saxton (G-S) Algorithm and Its Application,” the authors propose a series of modified G-S algorithms based on the Fresnel transform domain, including the single-phase retrieval (SPR) algorithm, the double-phase retrieval (DPR) algorithm, and the multiple-phase retrieval (MPR) algorithm. The analysis results show that the convergence of the SPR algorithm is better than that of the G-S algorithm, but the exact solution is not obtained. The DPR and MPR algorithms have good convergence and can obtain exact solutions; that is, the information is recovered losslessly. The authors discuss the security advantages and verification reliability of the proposed algorithms in image encryption. A multiple-image encryption scheme is proposed, in which n plaintexts can be recovered from n ciphertexts, which greatly improves the efficiency of the system. Finally, the proposed algorithms are compared with the current phase retrieval algorithms, and future applications are discussed. 

In the contribution by Anwar et al. [9], “An Image-Based Class Retrieval System for Roman Republican Coins,” authors propose an image-based class retrieval system for ancient Roman Republican coins that can be instrumental in various archaeological applications such as museums, numismatics study, and even online auction websites. For such applications, the aim is not only classification of a given coin, but also the retrieval of its information from a standard reference book. Such classification and information retrieval is performed by their proposed system via a user-friendly graphical user interface (GUI). The query coin image is matched with exemplar images of each coin class stored in the database. The retrieved coin classes are then displayed in the GUI along with their descriptions from a reference book. However, it is highly impractical to match a query image with each of the class exemplar images as there are 10 exemplar images for each of the 60 coin classes. Similarly, displaying all the retrieved coin classes and their respective information in the GUI will cause user inconvenience. Consequently, to avoid such brute-force matching, the authors incrementally vary the number of matches per class to find the least matches attaining the maximum classification accuracy. In a similar manner, the authors also extend the search space for coin class to find the minimal number of retrieved classes that achieve maximum classification accuracy. On the current dataset, their system successfully attains a classification accuracy of 99% for five matches per class, such that the top ten retrieved classes are considered. As a result, the computational complexity is reduced by matching the query image with only half of the exemplar images per class. In addition, displaying the top 10 retrieved classes is far more convenient than displaying all 60 classes.

The articles presented in this special issue provide insights into fields related to information entropy algorithms for image, video, and signal processing, including models, performance evaluation and improvements, and application developments. We hope the readers can benefit from the insights of these papers and contribute to these rapidly growing areas. We also hope that this special issue would shed light on major developments in the area of entropy and attract attention of the scientific community to pursue further investigations leading to the rapid implementation of these technologies.
